# Excessive urinary tract dilatation and proteinuria in pregnancy: a common and overlooked association?

**DOI:** 10.1186/1471-2369-14-52

**Published:** 2013-02-27

**Authors:** Giorgina B Piccoli, Rossella Attini, Silvia Parisi, Federica N Vigotti, Germana Daidola, Maria Chiara Deagostini, Martina Ferraresi, Agostino De Pascale, Francesco Porpiglia, Andrea Veltri, Tullia Todros

**Affiliations:** 1 Nephrology ASOU San Luigi Gonzaga, University of Turin, Turin, Italy; 2 Materno-Foetal Unit, OIRM S. Anna, University of Turin, Turin, Italy; 3 Radiology ASOU San Luigi Gonzaga, University of Turin, Turin, Italy; 4 Urology ASOU San Luigi Gonzaga, University of Turin, Turin, Italy

## Abstract

**Background:**

Proteinuria and dilatation of the urinary tract are both relatively common in pregnancy, the latter with a spectrum of symptoms, from none to severe pain and infection. Proteinuria is a rare occurrence in acute obstructive nephropathy; it has been reported in pregnancy, where it may pose a challenging differential diagnosis with pre-eclampsia.

The aim of the present study is to report on the incidence of proteinuria (≥0.3; ≥0.5 g/day) in association with symptomatic-severe urinary tract dilatation in pregnancy.

**Methods:**

Case series. Setting: Nephrological-Obstetric Unit dedicated to pregnancy and kidney diseases (January 2000-April 2011). Source: database prospectively updated since the start of the Unit. Retrospective review of clinical charts identified as relevant on the database, by a nephrologist and an obstetrician.

**Results:**

From January 2000 to April 2011, 262 pregnancies were referred. Urinary tract dilatation with or without infection was the main cause of referral in 26 cases (predominantly monolateral in 19 cases): 23 singletons, 1 lost to follow-up, 1 twin and 1 triplet. Patients were referred for urinary tract infection (15 cases) and/or renal pain (10 cases); 6 patients were treated by urologic interventions (“JJ” stenting). Among them, 11 singletons and 1 triple pregnancy developed proteinuria ≥0.3 g/day (46.1%). Proteinuria was ≥0.5 g/day in 6 singletons (23.1%). Proteinuria resolved after delivery in all cases. No patient developed hypertension; in none was an alternative cause of proteinuria evident. No significant demographic difference was observed in patients with renal dilatation who developed proteinuria versus those who did not. An association with the presence of “JJ” stenting was present (5/6 cases with proteinuria ≥0.5 g/day), which may reflect both severer obstruction and a role for vescico-ureteral reflux, induced by the stent.

**Conclusions:**

Symptomatic urinary tract dilatation may be associated with proteinuria in pregnancy. This association should be kept in mind in the differential diagnosis with other causes of proteinuria in pregnancy, including pre-eclampsia.

## Background

Dilatation of the urinary tract is common in pregnancy in particular in the last trimester [1-4]. The cause of the dilatation is disputed, some advocating hormonal effects and others obstruction. There is a broad spectrum of this ‘syndrome’, some women may be completely asymptomatic, others have only transient mild loin pain while other patients experience recurrent episodes of severe loin pain and/or lower abdominal pain; very occasionally these anatomical changes can be exaggerated with massive ureteral and renal pelvis distension (as well as slight reduction in renal cortical width) and very rarely, the changes may be extreme and precipitate a so-called “over-distension syndrome” even with reversible acute renal failure [5-7].

In general, dilatation on the right side is more common and more pronounced, being reported, in a mild form, usually involving calices only, in up to 90% of the pregnancies in the last gestational weeks. Near term, mild urinary dilatation tends to become bilateral, in keeping with the theory that it is caused by compression by the iliac arteries where the latter cross the true bony pelvis [8].

On the right side, dilatation is usually considered as presumably related to pregnancy, while dilatation on the left side, especially if unilateral, is probably related to a different situation and is less likely to resolve after delivery [5-8].

The possibility that urinary tract obstruction may occasionally cause proteinuria outside of pregnancy is well known, even if not frequently reported [9-11]; its degree is usually sub-nephrotic and its pathogenesis is usually related to increased pressure in the renal pelvis, leading to a diuretic response from the contralateral kidney. Proteinuria caused by kidney obstruction is usually mild and considered of tubular origin, although different mechanisms may be operating [12,13]. Very few cases of massive proteinuria in the context of unilateral renal dilatation have been reported. Notably, a relatively recent report dealt with a pregnant patient who developed nephrotic proteinuria. In this very well documented case, proteinuria was found to stem from both kidneys at the time of laparoscopic intervention and resolved after pyeloplasty performed a few months after delivery [14]. The authors of the report stated that the lack of proteinuria prior to pregnancy and the failure to resolve after delivery pointed to pregnancy as a catalyst in the development of proteinuria [14]. A few interesting cases had been reported, in which urinary tract dilatation was presumably the cause of severe pre-eclampsia or of kidney function impairment [15,16]. However, according to a Medline search at June 2011 (combining the terms proteinuria, hydronephrosis, kidney and dilatation with pregnancy), we could locate no surveys of the of the prevalence of proteinuria associated with urinary tract dilatation in pregnancy in the last decade, with the exception of the case mentioned above.

This issue is however of great interest, particularly with respect to the differential diagnosis with pre-eclampsia and other causes of pregnancy-induced proteinuria, including a vast array of chronic kidney diseases.

We therefore evaluated whether or not proteinuria without obvious cause was present in 26 pregnant women, referred to our specialty clinic with excessive and/or symptomatic urinary tract dilatation in pregnancy, with or without sign of upper urinary tract infection.

## Methods

### Study setting

The study was performed in the Maternal-Foetal Unit of the University Hospital OIRM S. Anna, Turin, Italy, where all pregnant patients with kidney diseases have been followed by the same obstetric and nephrological team since 2000. A large database had been structured and data were prospectively gathered since the start of the activity [17].

From January 2000 to April 30^th^ 2011, 262 pregnancies were observed in 235 women referred to our Unit dedicated to pregnancy and kidney diseases. Twenty-six of the patients (26 patients; 26 pregnancies) displayed excessive (above 3 cm) and/or symptomatic urinary tract dilatation (10% of the overall referred population) as a main clinical problem and reason for referral. The present analysis is focused on this cohort.

### Diagnostic and follow-up policies. Main definitions

The diagnostic work-up for patients with signs or symptoms of kidney disease referred to the Unit includes renal function assessment (at the first visit in the Unit: creatinine and proteinuria on 24-hour urine collection) and immunological and coagulation screening. Abdominal ultrasounds are performed at least once in all cases; they are routinely performed in the same two settings (Materno-Foetal Hospital and S Luigi Hospital) by a small group of skilled operators.

In the case of known urological problems preceding pregnancy, the follow-up policy includes urinalysis and urinary cultures every week, or on alternate weeks, as well as ultrasounds at least every three months, and more frequently in the case of urinary tract dilatation. Since the entity of the urinary tract dilatation is also dependent upon hydration and position, a clear cut-off between physiological and pathological was not available from the literature; for the present study, we considered as “excessive” the urinary tract dilatation when exceeding 3 cm of maximal diameter, or over 2 cm when symptomatic [1,6-8,18,19]. The dilatation was considered as symptomatic when accompanied by flank pain, enhanced by palpation, and or by signs of upper urinary tract infection.

Due to the risks of any invasive manoeuvre in pregnancy, positioning of “JJ stents” is limited to cases with severe dilatation, untreatable pain and/or evidence of complete or almost complete obstruction and infection.

Hypertension is defined as systolic blood pressure ≥140 and/or diastolic blood pressure ≥90, or anti-hypertensive therapy. In the case of hypertension, 24-hour blood pressure monitoring and echocardiography are performed; other analyses are prescribed on demand. CKD is classified according to K-DOQI guidelines, using pre-pregnancy data whenever possible. When these are not available, data at referral are used. The Cockcroft-Gault and EPI formulae (based upon the first available serum creatinine data) and 24-hour urine assessments (for the subsequent measurements) are routinely employed for the patients with CKD stages 2–5. For other details on definitions, please refer to our previous study [17].

The frequency of nephrological and obstetric controls is individualized, weekly to monthly. In addition to the routine pregnancy controls, creatinine clearance on 24 hour urine collection is routinely required at referral; all patients undergo (at least) a monthly determination of serum creatinine and dipstick proteinuria, uric acid, urinalysis and urinary culture, serum electrolytes, coagulation and blood cell counts. If proteinuria is present, 24-hour urine collection is requested. Other tests are required on demand. Proteinuria is classified into tubular pattern (exclusive or prevalent: Beta 2 microglobulin, alfa 2 microglobulin, lysozime), glomerular pattern (exclusive or prevalent: albumin, transferrin, IgG), and mixed pattern.

Ultrasound biometry and Doppler velocimetry of uterine and umbilical arteries are individualized (every two-four weeks if there is a risk of foetal growth restriction).

Hospitalization is required in the presence of uncontrolled or new-onset hypertension, worsening of renal function, new onset or worsening of proteinuria, or any intercurrent problem of mother and/or foetus (abnormal foetal growth and/or severely abnormal umbilical Doppler) [17].

Preterm delivery is defined as delivery before 37 completed weeks of gestation; “early pre-term” delivery is defined as delivery before the 34^th^ completed gestational week. Caesarean section is performed for foetal indications or in cases of unfavourable conditions for, or lack of response to, induction. Apgar scores are recorded at 1 and 5 min by the neonatologists. A newborn is defined as Small for Gestational Age (SGA) when the birth weight is below the 10^th^ centile according to Italian birth weight references [17].

### Patient selection

The patients were selected from the Unit’s database according to the following criteria: symptomatic or severe urinary tract dilatation at ultrasounds performed at any time during pregnancy; availability of at least one full functional evaluation with 24-hour proteinuria. In all cases, at least 2 controls of 24-hour proteinuria were available. Patients referred for acute upper urinary tract infection who did not display renal dilatation at ultrasounds were selected as a control group (14 cases, from the 262 pregnancies referred). After the initial selection, the clinical charts of all patients were reviewed by the same operators.

As the definition of “significant” proteinuria may be controversial, and some Authors stress the potential overlap between normal and pathological data, setting the limit at 0.3 g/day, two cut-points were tested: 0.3 and 0.5 g/day. In the patients who had developed proteinuria >=0.5 g/day, post-pregnancy data were obtained from the clinical charts or by phone inquiry.

### Ethical approval and consent to the study

The overall research and build-up of the archives of the patients followed in our Unit, focused on the differential diagnosis between pre-eclampsia and CKD and on the risks of CKD in pregnancy was approved by the Ethical committee of the OIRM-S. Anna Hospital of the University of Turin (number 335; protocol 11551/c28.2; data of final approval 4.3.2011); since the start of the activity, all patients followed in the Unit signed an informed consent for the use of anonymous data for research purposes. Specific consent was requested to the patient whose image is reported in the present paper.

### Statistical analysis

A descriptive analysis was performed as appropriate. The patients with renal dilatation and proteinuria were compared with the cases without proteinuria by Chi square test (discrete variables) and Student’s t-test (continuous variables). For non parametric comparisons, Mann Whitney test was employed.

## Results

### Prevalence of proteinuria in pregnant patients with urinary tract dilatation

In the study period (January 2000 - April 2011), 262 pregnancies in 235 women were referred to the Outpatient Unit for Kidney Diseases in Pregnancy. Symptomatic or excessive urinary tract dilatation was the main sign in 26 cases (10% of pregnancies): 23 singletons, 1 lost to follow-up, one twin, one triple pregnancy. Dilatation was predominantly monolateral in 19 cases.

The overall prevalence of proteinuria ≥0.3 g/day (the threshold level for the differential diagnosis with pre-eclampsia) associated with urinary tract dilatation was high: 11 singletons and 1 triple pregnancy developed proteinuria before term (46.1% of the overall population; 48% of the singletons, not considering the patient lost to follow-up).

Proteinuria was ≥0.5 g/day in 7 cases, namely 6 singletons and one triple pregnancy (26.9%).

Patients were mainly referred for upper urinary tract infection (15 cases) and/or renal pain (10 cases). In 4 cases, the finding of urinary tract dilatation exceeding 3 cm was incidental during an evaluation of kidney ultrasounds.

Six patients (5 of them in the higher proteinuria group) were treated by urologic interventions (“JJ” stenting), 4 in our institution and 2 in a different setting, before referral to our Unit.

### Characteristics of the patients with and without proteinuria

The main clinical and biochemical data of the patients with and without proteinuria (cutpoint at ≥0.3 g/day and ≥0.5 g/day) associated with urinary tract dilatation are reported in Table 1.

**Table 1:** Prevalence and main characteristics of patients, according to the presence of proteinuria, dilatation or infection (data at referral and delivery)

	**Patients with urinary tract dilatation and proteinuria <0.3 g/day (12 pts)**	**Patients with urinary tract dilatation and proteinuria ≥0.3 <0.5 g/day (5 pts)**	**Patients with urinary tract dilatation and proteinuria ≥0.5 g/day (6 pts)**	**Patients with acute urinary tract infection without dilatation (14 pts)**
**Mean age (start of pregnancy) ± sd**	28.6 ± 5.93	29.2 ± 5.93	29.8 ± 4.49	29.8 ± 6.60
**Primiparous n (%)**	5 (41.7%)	3 (60%)	2 (33.3%)	9 (64.3%)
**Median week of referral**	23 (4–36)	8 (5–33)	30 (18–35)	21 (7–36)
**Data at referral of pregnancy**				
**Mean creatinine (mg/dl) ± sd**	0.6 ± 0.12	0.7 ± 0.09	0.6 ± 0.21	0.6 ± 0.13
**GFR (EPI)**	124 ± 13	118 ± 12	125 ± 25	120 ± 14
**Hypertension**	No	No	No	No
Median proteinuria (g/24 h) *	0.1 (0.08-0.31)	0.1 (0.07-0.18)	1.09 (0.1-2.65)	0.1 (0.06-0.53)
**“JJ” stenting during pregnancy §**	0	1/5	5/6	0
**Data at delivery**				
**Mean creatinine (mg/dl) ± sd**	0.6 ± 0.15	0.6 ± 0.20	0.7 ± 0.22	0.6 ± 0.12
**GFR**	124 ± 18	118 ± 26	118 ± 27	117 ± 14
**Hypertension**	No	No	No	1 (7.1%)
median proteinuria (g/24 h) **	0.1 (0.07-0.16)	0.3 (0.3-0.38)	1.7 (0.6-2.54)	0.1 (0.1-0.23)
Mean gestational age ± sd ***	38.8 ± 1.7	38.6 ± 1.8	35.5 ± 1.8	38.8 ± 1.4
**Preterm delivery <37 weeks n (%)§§**	2 (16.7%)	0	4 (66.7%)	0
**Vaginal delivery** **n (%)**	10 (83.3%)	2 (40%)	6 (100%)	12 (85.7%)
Weight of newborn ± sd ****	3315.4 ± 548.92	3136 ± 453.02	2553.3 ± 372.86	3236.7 ± 494.7
**Newborns ≤10**^**th **^**centile**	0	0	1 (16.7%)	1 (7.1%)

Note: Singletons only; one twin and one triplet pregnancies in the group with dilatation were not considered. One further patient dropped out from follow-up before delivery (also excluded).

All patients underwent at least two measurements of proteinuria on 24 hour urine collections.

* Mann–Whitney test: group 3 versus 1: p= 0.002; versus 2: p=0.019; versus 4 p=0.012.

** Mann–Whitney test: group 3 versus 1: p= 0.000; versus 2: p=0.005; versus 4 p=0.000.

*** T student test: group 3 versus 1: p= 0.0016; versus 2: p=0.0184; versus 4 p=0.0003.

**** T student test: group 3 versus 1: p= 0.0077; versus 2: p=0.0437; versus 4 p=0.0074.

§ Chi square test: group 3 versus 1: p= 0.0016; versus 2: p=0.1356; versus 4 p=0.0007.

§§ Chi square test: group 3 versus 1: p= 0.1160; versus 2: p=0.0971; versus 4 p=0.0050.

Singletons only.

Due to the lack of referral data as for incidence of low-grade proteinuria in multiple pregnancies, only singletons are reported. One patient dropped out from follow-up before delivery, and was likewise excluded, thus leaving 23 cases for the statistical analysis.

No significant baseline difference was observed in patients with renal dilatation who developed proteinuria versus those who did not, nor in the cohort of patients referred for upper urinary tract infection without proteinuria (Table 1). Proteinuria always resolved after delivery and after stent removal, routinely performed within one month after delivery in all but one patient, in whom proteinuria tested negative at 3 months. No patient developed hypertension during pregnancy or in the first month after delivery; no alternative cause of proteinuria was evident. In keeping with the absence of other pathological conditions, delivery was vaginal in the vast majority of the cases in all subsets considered (against a background of 25% caesarean deliveries in low-risk pregnancies in our Unit [17]).

With regard to outcomes, patients with higher levels of proteinuria displayed significantly greater morbidity than those without proteinuria: delivery was preterm in 4/6 cases and thus a significantly lower birth weight was recorded (Table 1).

Interestingly, there was a strong association with the positioning of JJ stenting and proteinuria, as stenting had been performed in 5 cases with excessive renal dilatation and proteinuria and in only one case in the low-proteinuria group (Table 1).

### Characteristics of patients with proteinuria ≥0.5 g/day

The main clinical features and outcomes of singletons with proteinuria equal or above 0.5 g/day are summarised in Table 2. Only one patient (case 2) had a history of stone disease, although previously undetected kidney stones were found at ultrasounds in 3/5 cases. One patient had a history of urinary tract involvement, with colicky pain during a previous pregnancy. All patients had normal renal function throughout pregnancy and no ultrasound evidence of any condition predisposing to proteinuria or otherwise indicating “chronic kidney disease” (such as kidney scars or hyperechogeneity of the kidney parenchyma); no patient had signs or history compatible with vescico-ureteral reflux. No patient had evidence of viral infection (hepatitis B, C and HIV were negative in all cases), and all had normal “basic” immunological testing (complement, antinuclear factors, ENA, ANA, immunoglobulin levels). Proteinuria typing was performed in three patients; it revealed a mixed glomerular and tubular pattern with non-selective glomerular component in all three patients.

**Table 2:** Main reasons for referral and main clinical characteristics in singletons with ≥0.5 g/proteinuria/day and renal dilatation

	**Case 1**	**Case 2**	**Case 3**	**Case 4**	**Case 5**	**Case 6**
Age (start of pregnancy)	24	34	27	27	30	36
Weight (start of pregnancy) – Kg	68	59	64	55	65	50
BMI (start of pregnancy) Kg/m^2^	22.5	21.1	23.5	21.5	26	18.4
Parity	1011+1	1001	0000	1001	1001	0000
Week of referral	18+6	28	35+2	32+4	35+4	26+6
Serum Creatinine at referral	0.45	0.67	0.95	0.36	0.45	0.55
Stone disease (active)	Yes	Yes	No	Yes (brushite)	No	No
Stone disease (previous)	No	Yes	No	No	No	No
Urinary tract infection at referral	Yes	Yes	No	Yes	Yes	No
Urinary tract infection (history of)	No	Yes	No	No	No	No
Side of dilatation	Right (stenting); lesser degree left side	Left	Right	Left	Right	Right
Dilatation at assessment or stenting (cm)	4 cm	3 cm	3.5 cm	3.5 cm	2** cm	6.5 cm
JJ stenting (week) Reason for stenting	18^th^ week infection and ureteral stone	28^th^ week infection and ureteral stone	No	20^th^ week infection and ureteral stone	34^th^ week infection and severe pain	27^th^ week infection and severe pain
Maximum level of proteinuria	2.5 g/day	3.5 g/day	2.5 g/day	1.2 g/day	2.7 g/day	0.8 g/day
**Data at delivery**						
Gestational age	32+4	36 US	38	36+1	37+2	31+4
Weight gain - Kg	14	10	15	8	13	9
Type of delivery	vaginal	Vaginal	vaginal	vaginal	vaginal	vaginal
**Children:**	**Case 1**	**Case 2**	**Case 3**	**Case 4**	**Case 5**	**Case 6**
Sex	M	M	M	F	M	F
Weight	2400	2450	2760	2600	3110	2000
Centile	70^th^	15^th^	10^th^	30^th^	50^th^	60^th^
Apgar 5^′^	9/9	9/9	9/9	9/9	9/9	8/9

Note: **the patient was relatively de-hydrated at the first assessment; a second measurement after a few days of i.v. fluids and antibiotic therapy exceeded 3 cm. US adjusted: gestational age assessed with US datation.

**Figure 1:**
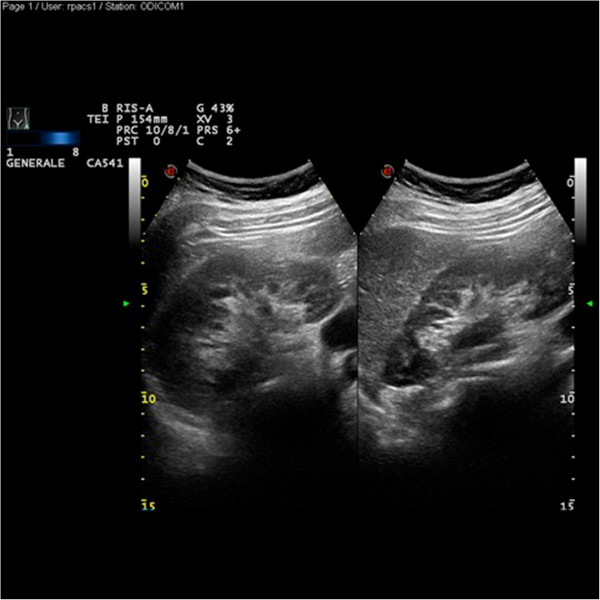
**Kidney ultrasounds in case 5, after the positioning of JJ stent.** Only minimal dilatation is present; however, proteinuria persisted after “JJ stenting”, reducing the dilatation, and in the absence of infection. “JJ” was removed one week after delivery (proteinuria 0.5 g/day); proteinuria was absent at the control one month after delivery.

In pregnancy, proteinuria was not limited to the phase of active infection in any patient, as it persisted or even developed (cases 4-5-6) after resolution of the acute infectious phase. Five patients underwent ureteral stenting, in 3 because of upper urinary tract infection and active stone disease, and in 2 because of severe infection and pain unresponsive to the conventional pain relievers (Table 2). Interestingly, the residual dilatation of the urinary tract was not correlated with the degree of proteinuria, which persisted even after obstruction was at least partially relieved by renal stenting (Figure 1, case 5). In no case proteinuria decreased after stenting, and in three patients it increased.

Stenting was a cause of significant morbidity in two cases: case 5 (urinary pain, urgency and frequency) and case 6, in which the invasive manoeuvre could have played a role in the preterm delivery (Table 2).

There was no evidence of an effect of the urological problem, the eventual infection or the therapies on the growth curves. In fact, the newborn was small for gestational age in only two cases, one in the subset with higher proteinuria and one in the control group with upper urinary tract infections without dilatation.

The urinary picture normalized in all patients within three months after delivery, with the disappearance of proteinuria; the ureteral stent was safely removed in all cases; it is impossible to disentangle the effect of ureteral stent removal from the effect of delivery, as the patients perform a limited number of 24 hour urine collections after delivery (usually 1 at 1 month, and - if proteinuria over 0.3 g/day is still present and decreasing - the test is repeated at 3 months).

## Discussion

The present report deals with an interesting association, not extensively studied thus far and possibly underreported, between symptomatic and/or excessive urinary tract dilatation and proteinuria in pregnancy. Indeed, our extensive Medline search revealed only one recently published case of an association between severe proteinuria, urinary tract dilatation and pregnancy in the last decade; a few other cases in which a pre-eclamptic syndrome was probably triggered by dilatation had been previously reported [13,15,20-22].

Our cases were less extensively studied than the one reported by Afzali and co-workers, also because proteinuria decreased below 0.3 g/day shortly after pregnancy in all, thus limiting the diagnostic potential which is higher after delivery [13]. Interestingly, in both our small series and the recently reported case, renal function was normal and no history of kidney disease (with particular reference to reflux nephropathy) was available nor was a different cause of kidney disease suggested by the ultrasound patterns. This differentiates our cases from reports of the risks of pregnancy in women with vescico-ureteral reflux in infancy, a potential risk factor even in the presence of normal kidney function [20-23].

It is well known that proteinuria may develop late in urological disease. This is usually considered to be caused by nephron loss and a poor prognostic sign, occurring in late stages of the disease when significant reduction of the kidney parenchyma is usually evident at ultrasounds [19,24,25]. However, neither our patients nor the case reported by Afzali displayed such a picture and the kidney function was normal in all cases. A “renal reflex” has been postulated in experimental animals and in humans, and was called into question in the case report by Afzali and co-workers [13,26,27].

It is very difficult to suggest a univocal interpretation of the observed data.

First of all, our cases were symptomatic or displayed excessive dilatation (over 3 cm); this referral pattern differentiates our observations from those of other studies, in which mild or minimal dilatations are reported, in the assessment of the frequency of urinary tract dilatation in pregnancy [24,25]. Thus, our series may be considered as negatively selected, as small symptomless dilatations are neither identified nor referred (the use of maternal kidney ultrasounds in pregnancy is very limited in the clinical practice). Furthermore, in the presence of proteinuria, ultrasounds are not routinely performed in all settings, and a relationship between the so-called pregnancy-induced proteinuria and non symptomatic urinary tract dilatation may escape diagnosis.

Therefore, we will limit our report to the description of an association that might be more frequent than previously reported, possibly because of a trigger effect of pregnancy on the development of proteinuria in the context of various kidney diseases, including symptomatic or severe urinary tract dilatation. As urinary picture normalized in all patients after delivery and ureteral stent removal; it was impossible to disentangle the effect of each ones. However, the clinical relevance in the differential diagnosis with preeclampsia is unaffected by the cause of proteinuria.

A role for the increase in abdominal pressure may be postulated, together with a facilitating role of the urinary tract infections, where present. However, possibly to the negative selection of the cases, mentioned above, a clear-cut relationship was not identified.

Pregnancy may facilitate the development of proteinuria in the context of severe-symptomatic urinary tract dilatation via the changes in the metabolic milieu, through hyperfiltration or both, thus suggesting to further investigate in these fields. One possible explanation is that proteinuria increases steadily in pregnant women as the levels of the soluble fms-like tyrosine kinase-1(sFlt-1) rise, whose effect on podocytes is to increase proteinuria. Patients with chronic interstitial nephritis might have reached the tubular maximum of reabsorbtion and display proteinuria near term, when circulating sFlt-1 is at its highest [28,29].

The striking association with ureteral stenting in our series needs further confirmation on a larger scale; indeed, there is a strong selection bias, as only the most symptomatic cases usually undergo invasive procedures. A role of iatrogenic vescico-ureteral reflux (linked to the presence of a ureteral stent) in the persistence of tubular damage (and/or of the inflammatory changes associated with urinary reflux and infection) can be postulated, but once again this awaits further confirmation in a prospective larger-scale analysis.

Our study has several limitations, partly shared by other observational studies in pregnancy: the problem of low grade proteinuria is very important in particular in a situation in which the upper physiological limits “touch” the limit for the definition of a severe disease (pre-eclampsia). Hence, we may have missed some cases with low-grade proteinuria and urinary tract dilatation, who tested negative at conventional urinalysis; conversely, the cases who tested positive at urinalysis or who were diagnosed with proteinuria at 24 hour urine collections were repeatedly controlled, thus ensuring against false positives (Tables 1, 2).

The interest in our report is mainly clinical, since it raises the hypothesis of an alternative source of proteinuria in a context in which pre-eclampsia is the most likely diagnosis. The clinical management would be different, for example the controversial “fluid management” often employed to offset the pre-eclamptic response may even be harmful in the context of urinary tract dilatation.

The treatment of the pregnant patient presenting with upper urinary pain and fever, or a kidney stone is quite obvious, but the differential diagnosis may be difficult in the absence of these symptoms. Hence, our case series suggests that an obstructive origin should be considered in the differential diagnosis of proteinuria in pregnancy, particularly in cases presenting without hypertension and with normal renal function. Further research in this field, with coordinated nephro-urological and gynaecological teams, is recommended.

## Conclusions

This report suggests considering urinary tract dilatation in the differential diagnosis of the new onset of proteinuria in pregnancy and, vice versa, systematically testing for proteinuria in pregnant patients with severe and symptomatic urinary tract dilatation. Our series also suggests a possible association between “JJ stenting” and the development of proteinuria, even though the association may be due to the negative selection of cases needing urological interventions.

This diagnosis could be very important in the differential diagnosis with other pregnancy-related conditions, such as pre-eclampsia. Greater awareness of this issue may help clarify the mechanisms underlying the development of proteinuria in the context of obstructive kidney diseases.

## Competing interests

The authors declare that they have no competing interests.

## Authors’ contributions

GBP, AV and TT designed the study; GBP and RA drafted the manuscript. FNV, GD and MCD participated to the management of the patients and performed the collection of the data of the patients in the Nephrology setting. SP took care of data collection and of the statistical analysis, supported by GD. ADP and AV carried out the ultrasound analysis. FP followed patients in the Urologic aspects. All authors read and approved the final manuscript.

## Pre-publication history

The pre-publication history for this paper can be accessed here:

http://www.biomedcentral.com/1471-2369/14/52/prepub

## References

[B1] Faúndes A Brícola-Filho M Pinto e Silva JL Dilatation of the urinary tract during pregnancy: proposal of a curve of maximal caliceal diameter by gestational age Am J Obstet Gynecol 1998 178 1082 1086 10.1016/S0002-9378(98)70552-6 9609588

[B2] Stothers L Lee LM Renal colic in pregnancy J Urol 1992 148 1383 1387 1433534 10.1016/s0022-5347(17)36917-3

[B3] Guichard G Fromajoux C Cellarier D Loock PY Chabannes E Bernardini S Maillet R Bittard H Kleinclauss F Management of renal colic in pregnant women, based on a series of 48 cases Prog Urol 2008 18 29 34 10.1016/j.purol.2007.11.001 18342153

[B4] Andreoiu M MacMahon R Renal colic in pregnancy: lithiasis or physiological hydronephrosis? Urology 2009 74 757 761 10.1016/j.urology.2009.03.054 19660792

[B5] Brown MA Urinary tract dilatation in pregnancy Am J Obstet Gynecol 1991 164 2 642 643 1992717 10.1016/s0002-9378(11)80039-6

[B6] Hladunewich M Odutayo A Thadhani R Coffman T, Falk R, Molitoris B, Neilson E, Schrier R The Normal and Diseased Kidney in Pregnancy Diseases of the kidney 2013 Wolters Kluwer: Lippincott Williams & Wilkins 1676 1709

[B7] Lindheimer MD Konrand KP Karumanki SA Renal Physiology and Disease in Pregnancy Seldin and Giebisch’s The Kidney 2008 Fourth USA: Elsevier INc 2339 2398 Physiology & Pathophysiology 1–2

[B8] Dure-Smith P Pregnancy dilatation of the urinary tract. the iliac sign and its significance Radiology 1970 96 3 545 550 5456311 10.1148/96.3.545

[B9] Klahr S Urinary tract obstruction Semin Nephrol 2001 21 133 145 10.1053/snep.2001.20942 11245776

[B10] Klahr S Morrissey J Obstructive nephropathy and renal fibrosis: The role of bone morphogenic protein-7 and hepatocyte growth factor Kidney Int Suppl 2003 82 S105 S112 14531782 10.1046/j.1523-1755.64.s87.16.x

[B11] Deng GY Sun JJ Wang P Mo JC Renal parenchymal thickness and urinary protein levels in patients with ureteropelvic junction obstruction after nephrostomy placement Int J Urol 2010 17 250 253 10.1111/j.1442-2042.2010.02448.x 20409217

[B12] Ware LB Johnson AC Zager RA Renal cortical albumin gene induction and urinary albumin excretion in response to acute kidney injury Am J Physiol Renal Physiol 2011 300 F628 F638 10.1152/ajprenal.00654.2010 21147844 PMC3064135

[B13] Afzali B Kingdon E Holt SG Treatment of unilateral obstruction reversing heavy and bilateral proteinuria Nephrol Dial Transplant 2005 20 210 212 10.1093/ndt/gfh575 15632352

[B14] Satin AJ Seiken GL Cunningham FG Reversible hypertension in pregnancy caused by obstructive uropathy Obstet Gynecol 1993 81 5 ( Pt 2) 823 825 8469485

[B15] Thorp JA Davis BE Klingele C Severe early onset preeclampsia secondary to bilateral ureteral obstruction reversed by stenting Obstet Gynecol 1999 94 5 Pt 2 806 807 10546735 10.1016/s0029-7844(99)00376-2

[B16] Nielsen FR Rasmussen PE Hydronephrosis during pregnancy: four cases of hydronephrosis causing symptoms during pregnancy Eur J Obstet Gynecol Reprod Biol 1988 27 3 245 248 10.1016/0028-2243(88)90129-3 3280354

[B17] Piccoli GB Attini R Vasario E Conijn A Biolcati M D’Amico F Consiglio V Bontempo S Todros T Pregnancy and chronic kidney disease: a challenge in all CKD stages Clin J Am Soc Nephrol 2010 5 844 855 10.2215/CJN.07911109 20413442 PMC2863984

[B18] Fried AM Woodring JH Thompson DJ Hydronephrosis of pregnancy: a prospective sequential study of the course of dilatation J Ultrasound Med 1983 2 6 255 259 6876256 10.7863/jum.1983.2.6.255

[B19] Rasmussen PE Nielsen FR Hydronephrosis during pregnancy: a literature survey Eur J Obstet Gynecol Reprod Biol 1988 27 3 249 259 10.1016/0028-2243(88)90130-X 3280355

[B20] Jungers P Houllier P Chauveau D Choukroun G Moynot A Skhiri H Labrunie M Descamps-Latscha B Grunfeld JP Pregnancy in women with reflux nephropathy Kidney Int 1996 50 593 599 10.1038/ki.1996.354 8840291

[B21] Jungers P Reflux nephropathy and pregnancy Ballieres Clin Obstet Gynaecol 1994 8 425 442 10.1016/S0950-3552(05)80329-3 7924016

[B22] Argueso LR Ritchey ML Boyle ET Jr Miliners DS Bergstrahl EJ Kramer SA Prognosis of children with solitary kidney after unilateral nephrectomy J Urol 1992 148 745 751 10.1016/s0022-5347(17)36710-1 1640559

[B23] Vu KH Van Dyck M Daniels H Proesmans W Renal outcome of children with one functioning kidney from birth. A study of 99 patients and a review of the literature Eur J Pediatr 2008 167 885 890 10.1007/s00431-007-0612-y 17940797

[B24] Cietak KA Newton JR Serial qualitative maternal nephrosonography in pregnancy Br J Radiol 1985 58 689 399 404 10.1259/0007-1285-58-689-399 3904901

[B25] Croce P Signorelli P Chiapparini I Dedè A Hydronephrosis in pregnancy. Ultrasonographic study Minerva Ginecol 1994 46 4 147 153 8065586

[B26] Golin R Genovesi S Stella A Zanchetti A Afferent pathways of neural reno-renal reflexes controlling sodium and water excretion in the cat J Hypert 1987 5 417 424 3668245

[B27] Protasoni G Golin R Genovesi S Zanchetti A Stella A Functional evidence of inhibitory reno-renal reflexes in spontaneously hypertensive rats Blood Press 1996 5 305 311 10.3109/08037059609078064 8879604

[B28] Reddy A Suri S Sargent IL Redman CW Muttukrishna S Maternal circulating levels of activin A, inhibin A, sFlt-1 and endoglin at parturition in normal pregnancy and pre-eclampsia PLoS One 2009 4 2 e4453 Epub 2009 Feb 11 10.1371/journal.pone.0004453 19412349 PMC2675175

[B29] Maynard SE Min JY Merchan J Lim KH Li J Mondal S Libermann TA Morgan JP Sellke FW Stillman IE Epstein FH Sukhatme VP Karumanchi SA Excess placental soluble fms-like tyrosine kinase 1 (sFlt1) may contribute to endothelial dysfunction, hypertension, and proteinuria in preeclampsia J Clin Invest 2003 111 5 649 658 12618519 10.1172/JCI17189 PMC151901

